# Personalized Dental Medicine: Impact of Intraoral and Extraoral Clinical Variables on the Precision and Efficiency of Intraoral Scanning

**DOI:** 10.3390/jpm10030092

**Published:** 2020-08-17

**Authors:** César Martínez-Rodríguez, Junco-Plana Patricia, Ortega-Aranegui Ricardo, Iglesias-Linares Alejandro

**Affiliations:** 1 Section of Post-Graduate Orthodontic, Faculty of Odontology, University Complutense of Madrid, 28001 Madrid, Spain; CESMARTINEZRODRIGUEZ@gmail.com (C.M.-R.); patalex71@hotmail.com (J.-P.P.); 2 Head Radiological Department, School of Dentistry, University of Madrid, 28001 Madrid, Spain; ricardoortega@odon.ucm.es; 3 BIOCRAN (Craniofacial Biology) Research Group, Complutense University, 28001 Madrid, Spain; 4 Full Professor of Orthodontics, Associate-Dean for Research, Complutense University of Madrid. Plaza Ramón y Cajal sn, 28001 Madrid, Spain

**Keywords:** scanners, orthodontics, malocclusion, digital acquisition

## Abstract

(1) Objectives: The aim is to measure the influence of different intraoral (crowding and molar inclination) and extraoral (surface material and ambient light) conditions on the efficacy and efficiency of intraoral scanning. (2) Methods: In a controlled in vitro experimental study, the samples were divided into six groups showing two types of intraoral conditions—lower incisor crowding (groups 1–3) and lower molar mesial tipping (groups 4–6). Each modified model was replicated using three types of materials with different light-absorption properties (*n* = 18 models). Each sample was scanned 30 times at light intensities of 0.0, 1800, or 3600 l×, yielding 3240 scans. Scanning efficiency (digital acquisition; scanning chair-time; and scanning failures) and scanning efficacy (undetected volume) were assessed using virtual superimpositions and Mecano Equate software. The intra- and interobserver error and reliability of the method were calculated and data analyses were performed using the *t*-test, paired *t*-test, and one-way analysis of variance (*p* < 0.05). (3) Results: Digital acquisition was influenced by the degree of crowding and molar inclination (*p* < 0.05). The scanning surface material affected the efficacy and efficiency, which were lower with a calcium sulfate hemihydrate A modified compound scanning surface (*p* < 0.05). Higher intensities of ambient light in the scanning room were associated with reduced scanning efficacy (*p* < 0.05). Moreover, the scanner showed greater amounts of undetected volume as the degrees of crowding and mesial tipping of the lower second molar increased over 25°, with mean error values of 0.97 mm^3^ and 1.12 mm^3^, respectively. (4) Conclusions: For scanning procedures employing digital acquisition, differences in the degrees of crowding and mesial tipping of the lower second molar, scanning surface material, and external light source intensity influence the efficacy and efficiency of the scanning procedures, scanning chair-time, scanning failures, and undetected volume.

## 1. Introduction

Intraoral scanners, one of the latest advancements in digital dentistry, have emerged as an alternative to conventional impressions that allow the development of complementary diagnostic tools and therapeutic procedures based on a digital workflow [1]. It has been shown [2] that intraoral scanning offers various advantages such as a more comfortable diagnostic record for the patient in comparison to traditional impressions, lower storage space requirement for casts, increased diagnostic versatility, greater precision, the chance to avoid reconstruction flaws such as voids and bubbles at critical regions of the impression, and generation of new therapeutic options [3].

Despite these obvious general advantages, some studies have noted limitations in the accuracy and reliability of intraoral scanning during complete scans of both arches [4,5,6]. Single tooth scanning shows greater accuracy than conventional impression methods [5], but as the scanning area increases, the precision of scanning tends to decrease under the influence of several factors [7]. In this regard, some factors, such as the intraoral scanning sequence, have been shown to affect the chair time and the accuracy of the records obtained [8], and other clinical conditions, such as the impact of different ambient lighting conditions can affect the accuracy of an intraoral scanning system as others have shown [9,10,11]. Moreover, various known and unknown factors can affect the accuracy of scanning capture and digital reconstruction, thus influencing the precision of the digital conversion of actual clinical information [10,11,12,13,14,15,16,17,18,19,20]. Nevertheless, the exact effects of intraoral or extraoral clinical conditions on the accuracy (efficacy) and efficiency of scanning have not yet been elucidated.

Therefore, the aim of this paper is (1) to quantify the influence of extraoral (light exposure at the scanning area (0.0, 1800, and 3600 lux), material-dependent light absorption of the scanned surface (high to low light absorption surfaces)) and (2) intraoral clinical conditions (crowding severity (2, 4, and 7 mm), magnitude of posterior tooth tipping (0°, 15°, and 30°)) on the accuracy (undetected volume (µm^3^) and efficiency of intraoral scanning (time, data size, and scanning failures).

## 2. Material and Methods

### 2.1. Study Design

**Figure 1:**
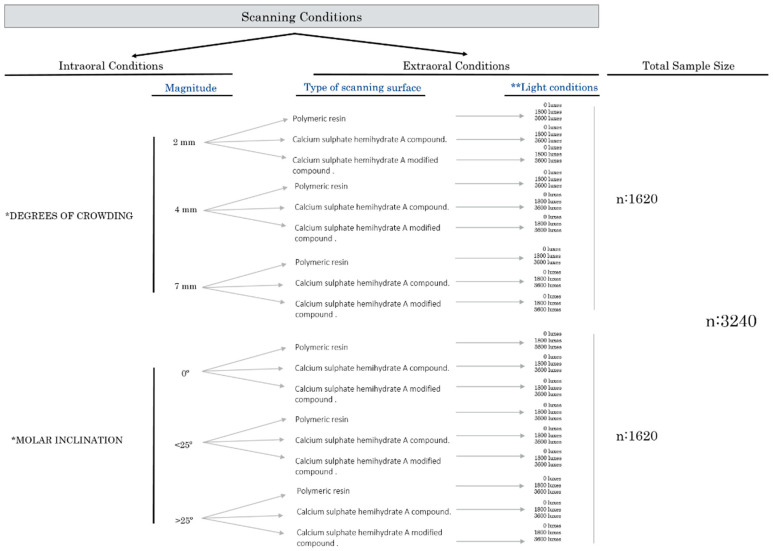
Scanning conditions. Two conditions were generated in a perfectly aligned plaster model, and the samples were divided into 6 groups. * Three groups for degree of crowding: G1, crowding of 2 mm by retroinclination of incisors; G2, crowding of 4 mm by rotating 2 teeth more than 25°; G3, crowding of 7 mm by rotating 4 teeth more than 25°. Three groups for molar inclination: G4, 2° premolar extraction of 7.2 mm with a molar inclination of 0°; G5, extraction of the first lower molar (10 mm) and the second molar inclination of 30°; G6, extraction of the first lower molar (10 mm) and the second molar inclination of 15°. ** Light conditions: each model was scanned under different light conditions measured with a luxmeter (Sekonic SE L308Sx): 0.0 l× (no light), 1800 l× (regular dental office light), 3600 l× (regular dental light office close to the window with sunlight).

A controlled in vitro experimental study was conducted. A detailed description of study groups, conditions, and experimental workflow is provided in Figure 1. All procedures and measurements were fully replicated by each of the two previously calibrated experienced operators, thereby ensuring the reliability of each step of the assessment process. None of the authors had any conflicts of interest related to the present study.

Briefly, using a perfectly aligned mandibular arch model as the digital master model (Supplementary Figure S1), six different controlled (in terms of magnitude and type) intraoral conditions were generated within the digital master model. Each new modified model was replicated with three types of materials with different light absorption optical properties—stereolithographic material (polymeric resin), calcium sulfate hemihydrate A compound, and calcium sulfate hemihydrate A modified compound—to yield a total of 18 models that were scanned 30 times each with the 3Shape TRIOS^®^ (3Shape Dental Systems, Copenhagen, Denmark) to obtain a total of 3240 scans (Figure 1). The intraoral conditions assessed were the degree of anterior crowding and molar inclination while the extraoral conditions were the intensity of light and the surface of the material scanned.

### 2.2. Ideal Cast, Virtual Set-Up Preparation, and Modified Cast Fabrication

#### 2.2.1. Ideal Cast Obtention (Control)

The ideal lower cast (control) was selected among 50 cases that had been orthodontically treated and finished correctly aligned with a 0-mm discrepancy without a fixed retainer, which ensured better scan quality. The bone-tooth discrepancy of the ideal cast was 0 mm, and the mesiodistal widths of all lower teeth were as follows: central incisors, 5 mm; lateral incisors, 5.5 mm; canines, 6.8 mm; first premolar, 7 mm; second premolar, 7.2 mm; first molar, 10 mm; and second molar, 9 mm. Thus, a scan of a perfectly aligned plaster lower cast served as the digital master model for the virtual set-up fabrication, which was performed with the intraoral modifications by using the Ortho-analyzer^TM^ software (3Shape, Copenhagen, Denmark).

#### 2.2.2. Virtual Set-Up Preparation: Intraoral Modifications (Crowding and Tipping)

**Figure 2:**
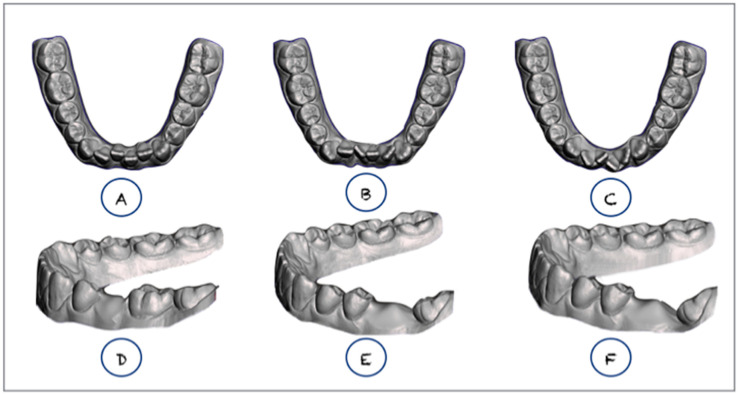
Intraoral conditions/Polymeric resin models. (**a**) G1: crowding of 2 mm; (**b**) G2: crowding of 4 mm; (**c**) G3: crowding of 7 mm; (**d**) G4: molar inclination of 0°; (**e**) G5: second molar inclination of 30°; (**f**) G6: second molar inclination of 15°.

Once the ideal cast was obtained, the intraoral modifications, crowding, and posterior tooth tipping were virtually created (Figure 2).

**Figure 3:**
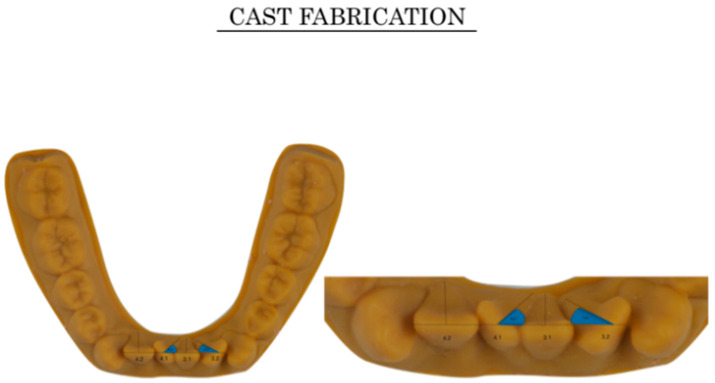
Different virtual set-ups. Degrees of crowding in the 3 groups: group 1 (G1): the model was fabricated by retroinclining the incisors until crowding of 2 mm was obtained; group 2 (G2): the model was generated by rotating two teeth more than 25° around their own long axial axis, i.e., exactly rotating the two lower incisors (tooth 3.2 and 4.1) by 65° to obtain crowding of 4 mm; group 3 (G3): the model was generated by rotating 4 incisors by more than 25°, i.e., exactly rotating tooth 3.2 and 4.1 by 70° and rotating tooth 3.1 and 4.2 by 30°, yielding a final crowding of 7 mm.

The first intraoral condition, degree of crowding, was virtually created by modification of the lingual inclination of the lower incisors while maintaining the canine and molar widths of the model, which allowed us to generate three models with different severities of crowding (Orthoanalyzer; 3Shape, Copenhagen, Denmark). Group 1 (G1): incisors were modified until 2-mm crowding was obtained; group 2 (G2): two lower incisors, 3.2 and 4.1, were rotated 65° around their own long axes to obtain 4 mm of crowding; and group 3 (G3): two lower incisors, 3.2 and 4.1, were rotated 70°, and 3.1 and 4.2 were rotated 30°, to yield a final crowding of 7 mm (Figure 3).

**Figure 4:**
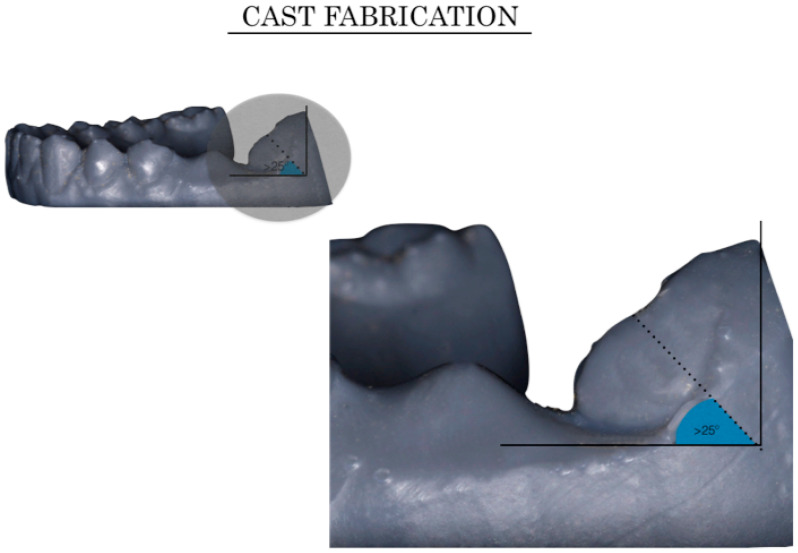
Different virtual set-ups. Molar inclination in the 3 groups as follows. Group 4 (G4): to generate the 4th model, we performed the extraction of the second lower premolar (tooth 3.5) whose mesiodistal width was 7.2 mm; group 5 (G5): the model was fabricated by extraction of the first lower molar (tooth 3.6) with a mesiodistal width of 10 mm while the second molar with a crown inclination (mesial tip) of 30°; group 6 (G6): was fabricated by extraction of the first lower molar (tooth 3.6) with a mesiodistal width of 10 mm while the second molar had a crown inclination (mesial tip) of 15°.

A second intraoral condition, mesial tipping of the lower second molar, was virtually reproduced as detailed in Figure 4. A virtual extraction was created mesial to the second lower molar and the second molar was mesially inclined 0° (Group 4, G4), 30° (Group 5, G5), or 15° (Group 6, G6) (Figure 4). The sample was thus divided into six groups under the four different intraoral conditions, respectively, with lower incisor crowding in groups 1–3 and lower molar mesial tipping in groups 4–6 (Figure 2).

#### 2.2.3. Extraoral Conditions (Cast Material and Light Conditions)

##### Scanning Surface Material

The ideal cast was 3D printed to reproduce six different virtual recreations by means of a Planmeca Creo printer (Planmeca, Helsinki, Finland), and the Formlabs 2 printer (Somerville, Massachusetts, USA). The calibration procedure of the 3D printers consisted of the printing of an STL file provided by the manufacturer, and after printing, a measurement was made with a caliber of that printed file, to subsequently introduce the measurements in the printer calibration program 3D. Printing models showed a flexible force (≥40 MPa), and the flexural modulus (≥1000 MPa) following the manufacturer’s recommendations, indicating that they are dimensionally stable materials.

In order to recreate different scanning surfaces and to determine whether the type of surface (differences in light absorption-reflection properties and differences in surface mechanical and optical properties) might influence the scanning procedure; three laboratory-controlled materials were used to create replicas of the six groups of intraoral conditions (G1–G6) with each of the three materials. Due to their influence on the reflection and refraction of light, we selected stereolithographic material (polymeric resin), calcium sulfate hemihydrate A compound, and calcium sulfate hemihydrate A modified compound (Figure 1). The clinical relevance lies in the fact that each of these materials has a different color so that the optical properties are different and the influence of these properties in the scanning process means the scanning might be affected.

To obtain poured impressions with the calcium sulfate hemihydrate A compound, we used a powder/water ratio of 100 g/27–28 mL at a working temperature of 68 °F with manual mixing for 60 s. The properties of this type of plaster were a setting expansion of 0.13%; hardness of 138 N/mm^2^ at 24 h; compressive strength of 35 N/mm^2^ after one hour; and white color. To obtain poured impressions with calcium sulfate hemihydrate A modified compound, we used a powder/water ratio of 100 g/22 mL with manual mixing for 60 s. The properties of this type of plaster were a setting expansion of 0.08%; compressive force of 35 N/mm^2^ after one hour; pink color [18].

##### External Light Source Intensity

The influence of external light exposure on the scanning procedure was evaluated. A luxmeter (Sekonic SE L308Sx) was used to determine the exact light intensity in the extraoral environment where scanning captures were performed. Three controlled luminous environments were created and evaluated: a completely dark room (0.0 l×), a room with the illumination levels of a conventional dental office (1800 l×) with artificial light, and a room with the illumination levels of a conventional dental office near a window, a source of natural light, and artificial light (3600 l×) [20].

In summary, a total of 18 models were used for the study (6 stereolithographic, 6 calcium sulfate hemihydrate A compound casts, and 6 calcium sulfate hemihydrate A modified compound), each of which was scanned 30 times in conditions involving no external illumination (0.0 l×), a standard-intensity external light source (1800 l×), and a high-intensity external light source (3600 l×).

### 2.3. Scanner and Scanning Method

Every cast was scanned with the 3Shape TRIOS^®^ (3Shape Dental Systems, Copenhagen, Denmark) intraoral scanner. The sequence recommended by the manufacturer was used; the scans were initiated in the occlusal area, followed by the lingual area, and finished in the vestibular area. The same sequence was reproduced in every cast, and the scanning caption was initiated within the fourth quadrant in all casts [13]. All procedures and measurements were fully replicated by each of the two calibrated experienced operators thereby ensuring the reliability of each step of the assessment process. A total of 3240 completed scanning files were obtained with this protocol (Figure 1).

### 2.4. Scanning Efficiency and Efficacy Evaluation

#### 2.4.1. Scanning Efficiency Assessment

The following measurements were assessed in every scan (Figure 1): (a) digital acquisition: for this parameter, the number of images obtained in each scan was determined. Once scanning was initiated, the 3Shape TRIOS^®^ software counted the number of images obtained until the complete digital model was completed. A greater number of images indicates greater difficulty in scanning the model or worse clinical conditions. (b) Scanning chair-time: in a previous pilot study (data not shown), the two operators scanned 100 patient models to complete their learning curve and avoid introducing biases. The scan times of all the models were obtained. A piece of equipment (Sekonic L-308S Flashmate^®^) that measures time accurately was used to measure the time taken to perform each scan, and the measurements were obtained in seconds up to two decimal digits (Figure 1). (c) Scanning failures: each time the scanner did not detect volume, image acquisition was stopped although the scan time continued, and the scanner returned to a previously registered area to continue. In order to determine scanning failures, the number of stops that occurred in each scan was quantified (Figure 1).

#### 2.4.2. Scanning Efficacy Assessment: Virtual Superimpositions

For analysis of the precision and accuracy of the scans, a modified version of the method proposed by Anh et al. (2018) was followed [19]. All the STL files of the different scans were superimposed over the original STL file with the Mecano Equate Program to determine the volume that the 3Shape TRIOS^®^ scanner could not detect, which was defined as (d) the undetected volume. The algorithm used in the virtual superimposition was about three points: the incisal edge of the incisors, the cusp of two molars, the first right molar, and the first left molar. These three points constitute a plane that is reproducible in all STL obtained from the different scans. This method was chosen due to it allowing us to identify the amount of scanned surface and those points that have not been registered during scanning and where there could be alterations when performing a prosthesisor an orthodontic aligner since they are sensitive areas that are not fully reproduced. The undetected volume was expressed in µm [3], and it provided an estimate of the volume that the scanner did not detect in the file and was subsequently artificially recreated by the scanner software, since if some of the cast volume is not detected there may be problems with fitting because the postproduction program that fills the undetected volume is not the real surface of the volume.

### 2.5. Statistics

#### 2.5.1. Intra-Interobserver Error and Reliability of the Method

All scans and measurements were duplicated by two independent operators, allowing the calculation of inter-examiner error. In addition, the intra-observer error was evaluated in over thirty random scans and thirty measurements were repeated by each examiner within a 3-week interval. Intra- and inter-examiner errors were assessed using the intraclass correlation coefficient (ICC) absolute agreement and the paired Student’s *t*-test. A *p*-value of less than 0.05 was considered to be statistically significant.

#### 2.5.2. Efficacy and Efficiency Assessments

Descriptive statistics (frequencies, percentage, mean, and standard deviation) were used to describe the distribution of the data in each group analyzed. The normality and homogeneity of the variables were tested by the Kolmogorov–Smirnov test. Thus, the variables used for efficacy and efficiency assessment (digital acquisition, scanning chair-time, scanning failures, and undetected volume) were evaluated using the *t*-test, paired *t*-test, and the one-way ANOVA. SPSS software version 25.0, (SPSS Inc., Chicago, IL, USA) was used for data analysis. The statistical significance level was set at a *p*-value of less than 0.05 (*p* < 0.05).

## 3. Results

### 3.1. Intra-Interobserver Error and Reliability of the Method

A total of 3240 measurements (1620 by each experienced examiner) were obtained. As shown in Table 1, intra-examiner (ICC: 0.89; *p* > 0.05) and inter-examiner error (ICC: 0.85 *p* > 0.05) were considered to be adequate, indicating good reliability of the method. Statistically significant differences were found in the efficacy and efficiency (digital acquisition, scanning chair-time, scanning failures, and undetected volume) of the scans due to the amount of crowding and the inclination of the molar to the edentulous gap (*p* < 0.05).

In assessments of the importance of the cast material, the quality and precision were reduced when calcium sulfate hemihydrate A modified cast was used (*p* < 0.005), indicating the influence of the scanning surface on these parameters. External lighting conditions also had a significant effect, with the precision at higher light intensities being lower (*p* < 0.05). Similarly, statistically significant differences were found in the digital acquisitions between the two operators (*p* < 0.005).

### 3.2. Impact of Intraoral Modifications (Crowding and Molar Inclination) and Extraoral Variations (External Light Intensity and Type of Material) on Scanning Efficiency

As shown in Table 1, with respect to the influence of the external source of light on the stereolithographic models, we found statistically significant differences at 3600 l× in terms of the number of images and scan time in crowded models (G1 (407.61 ± 53.68), G2 (464.32 ± 56.23), and G3 (469.06 ± 37.25)) (*p* < 0.005); in the absence of light, there were statistically significant differences in the time of scanning(s) both in the models with crowding and those with molar inclination (G1 (38.70 ± 6.66), G2 (47.24 ± 7.48), G3 (48.63 ± 12.02), G4 (41.75 ± 8), G5 (41.82 ± 7.89), and G6 (51.60 ± 5.8)) (*p* < 0.005).

As shown in Table 2, calcium sulphate hemihydrate A compound casts showed significant differences in terms of the number of images and scanning time at 3600 l× (G1 (378.03 ± 36.87), G2 (427.77 ± 43.89), G3 (438.12 ± 55.07), G4 (345.51 ± 32.56), G5 (437.48 ± 36.17), G6 (387.16 ± 34.70)) (*p* < 0.05); at 1800 l×, there were statistically significant differences in the number of images (G1 (318.70 ± 25.02), G2 (358.70 ± 38.25), G3 (364.32 ± 23.12), G4 (341.19 ± 24.60), G5 (439.03 ± 33.80), G6 (412.58 ± 29.62)) (*p* < 0.05)) and time of scanning(s) (G1 (32.20 ± 1.68), G2 (36.00 ± 1.69), G3 (36.00 ± 1.69), G4 (33.72 ± 1.90), G5 (44.33 ± 4.17), G6 (41.63 ± 4.59)) (*p* < 0.05). In addition, there were statistically significant differences in models with molar inclination (G4 (1.06 ± 0.72), G5 (2.16 ± 0.87), G6 (1.38 ± 1.11)) (*p* < 0.05) in terms of scanning failures.

Regarding the influence of light on the third type of material (calcium sulfate hemihydrate A modified compound casts) (Table 3), at 3600 l×, there were statistically significant differences (*p* < 0.05) in the casts with crowding in terms of the number of images (G1 (417.38 ± 53,36), G2 (499.45 ± 67.49), G3 (484.19 ± 70.17)) and time of scanning(s) (G1 (42.23 ± 3.56), G2 (51.91 ± 6.20), G3 (49.97 ± 5.28) (*p* < 0.05), but there were no statistically significant differences in cases of molar inclination. At 1800 l×, there were statistically significant differences (*p* < 0.05) in the number of images and undetected volume in the models with crowding (G1 (497.89 ± 95.46), G2 (560.70 ± 105.13), G3 (561.33 ± 124.53)) (*p* < 0.05). In the only group that showed statistically significant differences related to the inclination of the molar (G4 (45.57 ± 7.06), G5 (48.47 ± 8.30), G6 (48.05 ± 6.77) (*p* < 0.05)) and with this type of material cast, scanning was performed in the absence of an external source of light, and the only variable that showed statistically significant differences was scanning chair-time, with the rest of the variables not being statistically significant.

### 3.3. Impact of Intraoral Modifications (Crowding and Molar Inclination) and Extraoral Variations (Light Intensity and Type of Material) on Scanning Efficacy

The undetected volume (µm^3^) of the group exposed to 1800 l× with calcium sulfate hemihydrate A modified compound casts with crowding (G1: 0.06 ± 0.07; G2: 0.11 ± 0.09; G3: 0.26 ± 0.30) showed statistically significant differences in comparison with the other groups (*p* < 0.05). In the crowding models that were not exposed to an external source of light (G1, G2, and G3), we only found statistically significant differences in scanning chair-time(s) (G1: 38.63 ± 5.59; G2: 46.65 ± 6.52; G3: 49.24 ± 7.83) (*p* < 0.05); the molar inclination models also showed significant differences (*p* < 0.05) in the scanning chair time(s) (G4: 45.57 ± 7.06; G5: 48.47 ± 8.30; G6: 48.05 ± 6.77) (*p* < 0.05).

The undetected volume was comparable in all the groups except for the group with calcium sulphate hemihydrate A compound casts scanned at 3600 l× with the molar inclination variations. The mean undetected volume (µm^3^) of each group was as follows: G1, 0.07 ± 0.07; G2, 0.09 ± 0.09; G3, 0.1310 ± 0.14; G4, 0.16 ± 0.21; G5, 0.19 ± 0.27; G6, 0.18 ± 0.25 (*p* < 0.05).

To evaluate the precision of the scans, the volume that was not detected by the scanner was measured, and more scanning failures were obtained with severe crowding and an edentulous gap with a molar inclination higher than 25°, with error values of 0.97 µm^3^ and 1.12 µm^3^, respectively.

## 4. Discussion

The present study aimed to measure the influence of different extraoral (the type of material and light scanning exposure) and intraoral (degree of crowding and magnitude of molar inclination) clinical conditions on the efficacy and efficiency of intraoral scanning.

The application of digital models in different fields of dentistry has expanded exponentially recently, but there is still a lack of published scientific evidence that independently assesses the efficacy and efficiency of intraoral scans. Therefore, this study aimed to evaluate the influence of frequently encountered intraoral and extraoral conditions on both of these aspects by using a commercially available intraoral scanner.

Virtual superimposition methods are commonly used to assess the efficacy of intraoral scanning since they offer sufficient precision to accurately assess the coincidence of different digital images obtained from an intraoral scan [11,12]. Therefore, the precision in our study was evaluated by superimposing each generated STL image by placing three points in each of the casts for proper superposition. Some authors [13] have used alternative methods such as reference models, which involve creating a digital impression using a scannable abutment (for external-type implants, Scanbody, and for internal-type implants, impression healing abutment), generating STL files from the digital impression system, and importing them into a software to generate a color-coded map. Other authors have recently [8] used metal balls with a diameter of 1.5 mm that were placed at five locations of the model to evaluate the scanner accuracy, with superimpositions made by the Geomagic Verify TM software (3D Systems Inc; Rock Hill, SC, USA) to obtain a color map with the results.

Within the digital workflow, there are different types of intraoral scanners with different characteristics. Many authors have evaluated these devices. Therefore, previous studies that assessed the accuracy of 3Shape TRIOS^®^ in comparison with other intraoral scanners such as CEREC Omnicam or iTero [14] revealed some differences related to scanning-time, efficacy, and efficiency of intraoral scanning [15]. Thus, intraoral scanners are affected by different intraoral and extraoral clinical conditions that might influence the optimal conditions for intraoral scanning acquisition. Nevertheless, such potential influences have been not yet deeply tested and considered in studies assessing scanning accuracy, and thus, accuracy studies have been performed under different conditions, such as that by Yoon et al. [16], who evaluated the accuracy of intraoral scanning in patients with severe tooth crowding.

In the present study, we evaluated the accuracy of scanning within the context of increasing amounts of crowding and with an increasingly significant molar inclination. In particular, the G3 (crowding >7 mm) and G6 (molar inclination >25°) represented the most unfavorable clinical conditions examined, once the superpositions of all the models of the different clinical conditions described were performed. The results suggest that severe crowding and considerably inclined molars adjacent to an edentulous gap show statistically significant differences in comparison with the other conditions (Table 1, Table 2 and Table 3). One previous study [8] aimed to compare the accuracy of different 3D images obtained from different intraoral scanners and to evaluate the precision with different degrees of crowding and scanning sequences. The authors of that study used four models made with resin teeth and showed that crowding had no effect on the precision in the 3D model, in contrast to the results of the present study, wherein crowding was shown to affect the efficacy and efficiency of the scanning procedure. Therefore, a comparison of the two studies raises the question as to whether accurate and realistic digital impressions of scanning arcs with severe crowding can be obtained. In contrast, Yoo et al. [16] conducted a study on 58 patients divided into two groups depending on the degree of crowding to determine if digital scanning was more accurate than conventional methods. They did not observe statistically significant differences between digital and conventional methods. Despite the topic’s relevance, there exists a notable scarcity of research that establishes the clinical parameters to evaluate the effectiveness and efficiency of scanning. Other factors that could be critical, such as an increase in molar inclination, could reduce the efficacy and efficiency of intraoral scanning in situations of ambient, artificial light, and overexposure on various scanning surfaces [17]. Regarding the scanning sequence, we followed the manufacturer’s recommendations, since studies such as those by Ender et al. [7] comment that the scanning protocols that deviate from the manufacturer’s recommendation showed a significantly lower precision.

Some authors have compared the accuracy of scanning between different intraoral scanners, showing that the results obtained with the TRIOS scanner in in vitro studies are more accurate than those obtained with other intraoral scanners such as iTero (Cadent, Align Technology), CEREC systems (Bluecam and Omnicam, Sirona Dental Systems), and Lava COS (3M Espe) [12,18]. Nevertheless, the present study intended to compare the efficacy and efficiency of this intraoral scanner in different controlled intra- and extraoral conditions, including different external sources of light and different scanning surfaces. The intraoral scanner used in the present study, 3Shape TRIOS^®^, uses the principle of confocal parallel microscopy [19], which is based on eliminating the reflected or fluorescent light emitted from the planes out of focus. The use of a laser as a light source allows us to focus the illumination in a very small region of the sample and with great intensity. The results from the present study have shown that the ambient light is a critical factor to consider when performing an intraoral scan because it causes a defect in the scanning procedure and a delay in capturing the image [20]. Thus, we could observe that the light conditions have a statistically significant effect, resulting in lower precision, longer scan time, and more scanning failures with higher light intensities in the scanning room (*p* < 0.05). These results are similar to those obtained by Arakida et al. [21] in similar research that studied the influence of light and the time of exploration in the intraoral scan. Moreover, the results are in line with the research that Revilla et al. [9], who showed that ambient lighting conditions significantly influenced the scanning accuracy of the IOS system evaluated.

Therefore, our study reveals that the adequate lighting for digital scanning stereolithographic models is that of a conventional standard dental office (1800 lux), with the other models not showing statistically significant differences.

Regarding the influence of the type of surface to be scanned and the material cast on the intraoral scan efficiency, there is a scarcity of research in the literature. The calcium sulfate hemihydrate A modified compound scanning surface showed decreased scanning efficiency considering the digital acquisition variable (statistically significant in every group in plaster type III and only statistically significant in crowding 3600 l× and 1800 l× groups), which might be partially explained by the fact that the surface of calcium sulfate hemihydrate A compound casts showed a greater amount of light reflection, and they have a more polished surface than the modified compound material [22]. This finding highlights the substantial importance of surface type as a source of potential error in scanning, thus indicating that the capture properties of the scanner might be affected by the type of surface to be scanned, which may be extrapolated to different types of dental or gingival surfaces, saliva composition and retention on dental surfaces, adhesion of other organic/inorganic compounds to the dental surfaces, and other influencing factors.

The present research, however, is not exempt from limitations. This study offers novel data regarding the influence of specific controlled variables in the scanning process, under in vitro conditions, which might not be extrapolated to the in vivo clinical scenario directly. Nevertheless, intraoral scanning in the patient’s mouth might be exposed to different factors (i.e., light and dark zones) that might not be exactly reproduced in the in vitro experiments and that might represent additional sources of error or inaccuracy during scanning. The results from the present research should be further tested in an *in vivo* research, to confirm to which extent these factors and others might influence the accuracy and efficiency of the scanning process.

Since digital orthodontics is becoming exponentially available and is clinically useful, it is critical to determine the influence of all potential clinical factors that might affect the quality of diagnostic and therapeutic records offered by currently available intraoral scanners. To the best of our knowledge, the present research is the first to precisely quantify and report the impact of specific clinical intraoral and extraoral factors on the efficacy and efficiency of an intraoral scanner.

## 5. Conclusions

On the basis of the findings, the following conclusions can be drawn:

1. Clinical conditions such as tooth irregularities, molar inclination, cast material, and light conditions affect the scanning efficiency and can modify the digital acquisition, scanning chair-time, and scanning failures, taking into consideration that it is a technique-dependent procedure;
2. There are more scanning failures in cases with severe crowding and greater molar inclination than in other internal conditions, necessitating more images and greater chair-time;
3. Evaluation of the efficacy of scanning showed that the degree of inaccuracy influenced by intra and extraoral factors might have a negative impact on daily clinical practice;
4. Future in vivo randomized clinical trials should be conducted in order to determine to what extent these and other clinical factors might be influencing the scanning process and its results.

## Figures and Tables

**Table 1:** Efficiency and efficacy of scanner under variable intraoral (G1–G6) and extraoral conditions (light) with scanning surface of polymeric resin.

**3600 luxes (artificial light + natural light)**								
	**Group 1**	**Group 2**	**Group 3**	***p* value ¶**	**Group 4**	**Group 5**	**Group 6**	***p* value ¶**
Digital adquision	407.61 ± 53.68	464.32 ± 56.23	469.06 ± 37.25	0.001 **	441.33 ± 42.41	475.90 ± 26.29	467.80 ± 38.34	0.034
Scanning chair time (s)	42.04 ± 4.19	48.54 ± 6.63	46.03 ± 3.86	0.001 **	50.56 ± 3.01	51.23 ± 3.14	50.68 ± 1.72	1.002
Scanning failures	0.64 ± 0.60	1.25 ± 0.99	0.96 ± 1.01	1.002	1.50 ± 1.10	1.45 ± 0.99	1.22 ± 0.88	0.011
Undetected volume (µm^3^)	0.02 ± 0.03	0.03 ± 0.07	0.08 ± 0.14	1.003	0.05 ± 0.08	0.05 ± 0.08	0.05 ± 0.08	1.001
**1800 luxes (artificial light)**								
	**Group 1**	**Group 2**	**Group 3**	***p* value ¶**	**Group 4**	**Group 5**	**Group 6**	***p* value ¶**
Digital adquision	369.09 ± 69.03	418.03 ± 47.75	458.90 ± 88.73	0.022	416.90 ± 57.87	471.67 ± 108.06	449.12 ± 40.41	1.001
Scanning chair time (s)	36.58 ± 5.72	45.31 ± 6.47	47.89 ± 7.77	0.087	43.09 ± 7.34	46.68 ± 8.48	46.31 ± 5.91	1.001
Scanning failures	0.67 ± 0.70	1.06 ± 1.12	1.19 ± 0.87	1.001	1.13 ± 1.10	1.38 ± 0.88	1.48 ± 1.12	1.001
Undetected volume (µm^3^)	0.01 ± 0.01	0.03 ± 0.06	0.09 ± 0.19	1.002	0.09 ± 0.20	0.16 ± 0.20	0.11 ± 0.35	1.002
**0 luxes (no light)**								
	**Group 1**	**Group 2**	**Group 3**	***p* value ¶**	**Group 4**	**Group 5**	**Group 6**	***p* value ¶**
Digital adquision	434.61 ± 59.85	432.96 ± 69.67	437.93 ± 85.53	1.001	428.16 ± 68.41	406.61 ± 65.43	439.67 ± 45.37	1.001
Scanning chair time (s)	38.70 ± 6.66	47.24 ± 7.48	48.63 ± 12.02	0.001 **	41.75 ± 8	41.82 ± 7.89	51.60 ± 5.8	0.001 **
Scanning failures	1.06 ± 0.99	1.70 ± 1.18	1.80 ± 1.30	0.426	1.43 ± 1.35	1.70 ± 1.10	1.51 ± 0.88	1.002
Undetected volume (µm^3^)	0.096 ± 0.90	0.14 ± 0.14	0.10 ± 0.18	1.002	0.07 ± 0.08	0.16 ± 0.20	0.11 ± 0.10	1.001

G1: crowding of 2 mm; G2: crowding of 4 mm; G3: crowding of 7 mm; G4: Molar inclination of 0°; G5: Second molar inclination of 30°; G6: Second molar inclination of 15°; Digital acquisition: the number of images obtained for each scan; Scanning chair-time: time is needed to scan a model; Scanning failures: each time the scanner does not detect volume; Undetected volume: estimate of how much volume the scanner does not detect; ** *p* < 0.001; ¶ *p*: Bonferroni.

**Table 2:** Efficiency and efficacy of scanner under variable intraoral (G1–G6) and extraoral conditions (light) with scanning surface of calcium sulphate hemihydrate A compound.

**3600 luxes (artificial light + natural light)**								
	**Group 1**	**Group 2**	**Group 3**	***p* value ¶**	**Group 4**	**Group 5**	**Group 6**	***p* value ¶**
Digital adquision	378.03 ± 36.87	427.77 ± 43.89	438.12 ± 55.07	0.001 **	345.51 ± 32.56	437.48 ± 36.17	387.16 ± 34.70	0.001 **
Scanning chair time (s)	37.87 ± 3.43	42.02 ± 3.81	42.29 ± 4.95	0.001 **	34.51 ± 2.95	43.70 ± 3.34	39.34 ± 2.76	0.001 **
Scanning failures	1.77 ± 0.99	1.80 ± 1.01	2.29 ± 0.97	1	1.51 ± 0.96	2.09 ± 0.87	1.45 ± 0.50	0.183
Undetected volume (µm^3^)	0.08 ± 0.08	0.11 ± 0.10	0.16 ± 0.20	1	0.16 ± 0.25	0.17 ± 0.24	0.25 ± 0.25	0.023
**1800 luxes (artificial light)**								
	**Group 1**	**Group 2**	**Group 3**	***p* value ¶**	**Group 4**	**Group 5**	**Group 6**	***p* value ¶**
Digital adquision	318.70 ± 25.02	358.70 ± 38.25	364.32 ± 23.12	0.001 **	341.19 ± 24.60	439.03 ± 33.80	412.58 ± 29.62	0.002 *
Scanning chair time (s)	32.20 ± 1.68	36.00 ± 1.69	36.00 ± 1.69	0.001 **	33.72 ± 1.90	44.33 ± 4.17	41.63 ± 4.59	0.001 **
Scanning failures	0.61 ± 0.66	1.35 ± 0.55	1.58 ± 0.67	0.006	1.06 ± 0.72	2.16 ± 0.87	1.38 ± 1.11	0.003 *
Undetected volume (µm^3^)	0.08 ± 0.06	0.13 ± 0.09	0.19 ± 0.20	1.002	0.033 ± 0.10	0.06 ± 0.20	0.052 ± 0.099	1.001
**0 luxes (no light)**								
	**Group 1**	**Group 2**	**Group 3**	***p* value ¶**	**Group 4**	**Group 5**	**Group 6**	***p* value ¶**
Digital adquision	348.45 ± 28.33	373.77 ± 24.12	466.45 ± 53.20	0.001 **	327.03 ± 23.58	393.96 ± 40.74	428.74 ± 38.93	0.003 *
Scanning chair time (s)	34.52 ± 2.39	37.62 ± 2.36	45.06 ± 3.33	0.001 **	32.61 ± 1.63	39.92 ± 3.39	42.14 ± 2.60	0.002 *
Scanning failures	0.93 ± 0.77	1.64 ± 0.66	1.96 ± 0.83	0.001 **	0.96 ± 0.70	2.58 ± 1.08	1.83 ± 0.58	0.002 *
Undetected volume (µm^3^)	0.07 ± 0.07	0.09 ± 0.092	0.13 ± 0.14	1.002	0.16 ± 0.21	0.19 ± 0.27	0.18 ± 0.25	1.001

G1: crowding of 2 mm; G2: crowding of 4 mm; G3: crowding of 7 mm; G4: Molar inclination of 0°; G5: Second molar inclination of 30°; G6: Second molar inclination of 15°; Digital acquisition: the number of images obtained for each scan; Scanning chair-time: time is needed to scan a model; Scanning failures: each time the scanner does not detect volume; Undetected volume: estimate of how much volume the scanner does not detect; * *p* < 0.005; ** *p* < 0.001; ¶ *p*: Bonferroni.

**Table 3:** Efficiency and efficacy of scanner under variable intraoral (G1–G6) and extraoral conditions (light) with scanning surface of calcium sulphate hemihydrate A modified compound.

**3600 luxes (artificial light + natural light)**								
	**Group 1**	**Group 2**	**Group 3**	***p* value ¶**	**Group 4**	**Group 5**	**Group 6**	***p* value ¶**
Digital adquision	417.38 ± 53.36	499.45 ± 67.49	484.19 ± 70.17	0.001 **	456.43 ± 60.61	487.25 ± 29.51	481.06 ± 42.42	0.219
Scanning chair time (s)	42.23 ± 3.56	51.91 ± 6.20	49.97 ± 5.28	0.001 **	52.24 ± 3.63	52.66 ± 4.18	52.76 ± 2.67	1.005
Scanning failures	1 ± 0.68	1.90 ± 1.39	1.22 ± 1.17	0.831	1.80 ± 1.15	1.58 ± 0.99	1.61 ± 1.11	0.367
Undetected volume (µm^3^)	0.076 ± 0.084	0.091 ± 0.10	0.19 ± 0.22	0.212	0.15 ± 0.14	0.20 ± 0.24	0.21 ± 0.24	1.001
**1800 luxes (artificial light)**								
	**Group 1**	**Group 2**	**Group 3**	***p* value ¶**	**Group 4**	**Group 5**	**Group 6**	***p* value ¶**
Digital adquision	497.89 ± 95.46	560.70 ± 105.13	561.33 ± 124.53	0.002 *	420.36 ± 65.71	428.16 ± 86.98	419.45 ± 71.73	0.847
Scanning chair time (s)	56.31 ± 10.48	62.24 ± 14.04	60.36 ± 12.84	0.016	46.68 ± 12.31	48.59 ± 7.63	46.60 ± 10.40	1.003
Scanning failures	2.10 ± 1.37	2.23 ± 1.27	2.40 ± 1.32	1.001	2.06 ± 1.87	1.83 ± 1.34	2.25 ± 1.56	0.688
Undetected volume (µm^3^)	0.06 ± 0.07	0.11 ± 0.09	0.26 ± 0.30	0.002 *	0.15 ± 0.12	0.23 ± 0.20	0.21 ± 0.26	1.001
**0 luxes (no light)**								
	**Group 1**	**Group 2**	**Group 3**	***p* value ¶**	**Group 4**	**Group 5**	**Group 6**	***p* value ¶**
Digital adquision	385.48 ± 63.14	431.20 ± 48.98	473.77 ± 84.37	1.001	438.60 ± 59.00	478.67 ± 88.32	471.32 ± 43.48	1.002
Scanning chair time (s)	38.63 ± 5.59	46.75 ± 6.52	49.24 ± 7.83	0.002 *	45.57 ± 7.06	48.47 ± 8.30	48.05 ± 6.77	0.005 *
Scanning failures	0.77 ± 0.99	1.41 ± 1.28	1.67 ± 1.30	0.606	1.23 ± 1.25	1.70 ± 1.07	1.61 ± 1.20	1.002
Undetected volume (µm^3^)	0.09 ± 0.06	0.11 ± 0.92	0.18 ± 0.22	0.612	0.070 ± 0.0733	0.21 ± 0.25	0.22 ± 0.311	0.072

G1: crowding of 2 mm; G2: crowding of 4 mm; G3: crowding of 7 mm; G4: Molar inclination of 0°; G5: Second molar inclination of 30°; G6: Second molar inclination of 15°; Digital acquisition: the number of images obtained for each scan; Scanning chair-time: time is needed to scan a model; Scanning failures: each time the scanner does not detect volume; Undetected volume: estimate of how much volume the scanner does not detect; * *p* < 0.005; ** *p* < 0.001; ¶ *p*: Bonferroni.

## Supplementary Materials

The following are available online at https://www.mdpi.com/2075-4426/10/3/92/s1, Figure S1: Ideal cast. Perfectly aligned model (.STL) with a 0-mm discrepancy, without a fixed retainer from Ortho analyzer^TM^ (3Shape, Copenhagen, Denmark) software.
