# Supplementary figures and images for: Personalized Dental Medicine: Impact of Intraoral and Extraoral Clinical Variables on the Precision and Efficiency of Intraoral Scanning
Source: J Pers Med. 2020 Aug 17;10(3):92. doi: 10.3390/jpm10030092 (PMC7565800; doi:10.3390/jpm10030092)

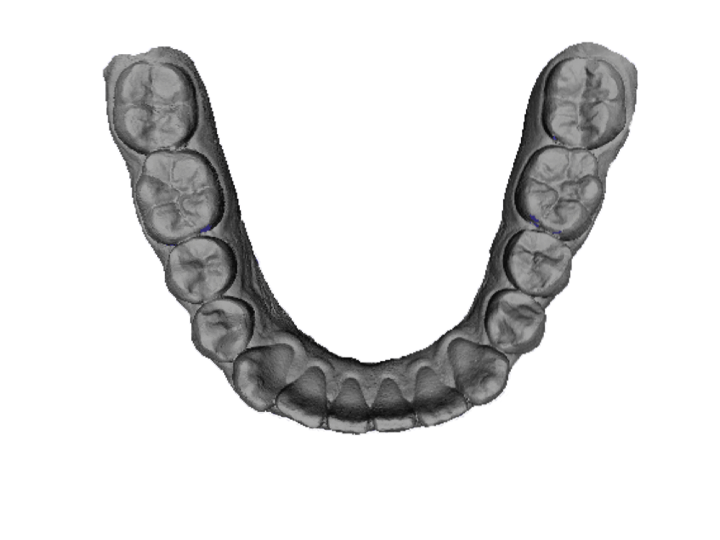

Supplement: Supplementary file 1 [file jpm-10-00092-s001.zip › jpm-893186-supplementary.tiff]
